# Benzodiazepines interfere with the efficacy of pembrolizumab-based cancer immunotherapy. Results of a nationwide cohort study including over 50,000 participants with advanced lung cancer

**DOI:** 10.1080/2162402X.2025.2528955

**Published:** 2025-07-04

**Authors:** Léa Montégut, Adrien Rousseau, Cinzia Ungolo, Lisa Derosa, Marine Fidelle, Carolina Alves Costa Silva, Bertrand Routy, Laurence Zitvogel, Benjamin Besse, Edoardo Pasolli, Guido Kroemer

**Affiliations:** a Centre de Recherche des Cordeliers, Equipe labellisée par la Ligue contre le cancer, Inserm U1138, Université Paris Cité, Sorbonne Université, Paris, France; b Metabolomics and Cell Biology Platforms, Gustave Roussy Institut, Villejuif, France; c Department of Cancer Medicine, Gustave Roussy, Thoracic Group and International Center for Thoracic Cancers (CICT), Paris-Saclay University, Villejuif, France; d Oncostat U1018, Inserm, Labeled Ligue Contre le Cancer, Paris-Saclay University, Villejuif, France; e INSERM U1015, Equipe Labellisée-Ligue Nationale contre le Cancer, Gustave Roussy Cancer Campus, Université Paris-Saclay, Villejuif, France; f Axe cancer, Centre de Recherche du Centre Hospitalier de l’Université de Montréal (CRCHUM), Montréal, Canada; g Hemato-Oncology Division, Centre Hospitalier de l’Université de Montréal (CHUM), Montréal, Canada; h Department of Biology, Center of Clinical Investigations in Biotherapies of Cancer (CICBT) BIOTHERIS, Villejuif, France; i Department of Agricultural Sciences, University of Naples Federico II, Portici, Italy; j Department of Biology, Institut du Cancer Paris CARPEM, Hôpital Européen Georges Pompidou, AP-HP, Paris, France

**Keywords:** ACBP, acyl CoA binding protein, NSCLC, lung adenocarcinoma, lung squamous carcinoma, psychotropic agents, benzodiazepines

## Abstract

We previously reported that elevated levels of diazepam binding inhibitor (DBI), also called ‘endozepine’ because it acts as an endogenous benzodiazepine equivalent on the gamma-aminobutyric acid type A receptor, constitutes a potential risk factor for the diagnosis of non-small cell lung cancer (NSCLC). Antibody-mediated neutralization of DBI improved the immunosurveillance of NSCLC in preclinical models with and without immunotherapy targeting programmed cell death protein 1 (PD-1). A pilot study in a small French-Canadian cohort (*n* = 205) suggested that benzodiazepine (BZD) use correlates with reduced progression-free survival in NSCLC patients receiving PD-1/PD-L1 blockade. Here, we report a retrospective analysis of the nation-wide French registry of advanced NSCLC patients treated with pembrolizumab. Among the eligible NSCLC patients surviving ≥2 months after treatment initiation (*n* = 31,479), 37.7% (*n* = 11,878) received at least two prescriptions of benzodiazepines within 90 days before to 30 days after treatment initiation. Compared to non-users (*n* = 19,601), BZD users had significantly reduced overall survival (hazard ratio = 1.08, 95% CI: 1.04–1.12, *p* < 0.001), an effect that persisted after correction using inverse probability of treatment weighting (IPTW) on sociodemographic, clinical, oncologic, and comedication variables. In a subset of 556 patients from the ONCOBIOTICS study, benzodiazepine use was associated with signs of intestinal dysbiosis and alterations in the TOPOSCORE, a prognostic marker linked to poorer outcomes in cancer patients receiving immunotherapy. We conclude that benzodiazepine use may be an independent negative prognostic factor for NSCLC patients under pembrolizumab-based immunotherapy. Future studies must determine whether withdrawal of benzodiazepines or neutralization of DBI improves the clinical response to immunotherapy.

## Introduction

Mounting evidence indicates that one quintessential mechanism of oncogenesis and tumor progression is the failure of cancer immunosurveillance.^1–3^ Accordingly, treatments designed to enhance the anticancer immune response are now occupying a central stage in the clinical management of multiple cancer types including non-small cell lung cancer (NSCLC),^4–6^ which is one of the most frequent malignancies affecting both men and women.^7^ In France, patients with advanced, inoperable NSCLC expressing high PD-L1 levels (on ≥50% of the malignant cells) receive the anti-PD1 monoclonal antibody (mAb) pembrolizumab, while patients expressing lower PD-L1 levels (<50%) receive pembrolizumab together with chemotherapy. A close-to-comprehensive analysis of the French population, the ATHENA cohort,^8^ has confirmed the efficacy of pembrolizumab irrespective of treatment duration (< or ≥2 years) and has unraveled covariables that were independently associated with worse overall survival (OS), including male sex with chemo-immunotherapy, advanced age, treatment in hospitals with low recruitment of NSCLC patients, high deprivation index, inpatient hospitalization for the first pembrolizumab treatment, a history of diabetes, or prescription of diuretics, beta blockers, or painkillers.^8^ These observations support the idea that sociodemographic features, the general health state, and perhaps comedications affect the efficacy of immunotherapy in NSCLC patients, likely by modulating the anticancer immune response.^9^ Among the potential mechanisms, growing evidence suggests that shifts in microbiota composition – often associated with poor health and aging, a condition known as ‘dysbiosis’^10–12^– may influence NSCLC immunotherapy outcomes. In an effort to define clinically relevant signatures,^13^ we demonstrated that an increase in harmful fecal bacteria from the unfavorable species-interacting group (SIG) SIG1, a decrease in beneficial bacteria from the favorable group SIG2, or any deviation from the optimal relative abundance of *Akkermansia* species, as epitomized in the TOPOSCORE, could predict the success of immunotherapy in NSCLC.^14,15^ Recently, we also identified the elevation of the circulating levels of diazepam binding inhibitor (DBI, also known as acyl CoA binding protein, ACBP, or ‘endozepine’) as a biomarker of biological aging.^16,17^ The plasma concentration of DBI does not only increase with chronological aging^18^ but also correlates with cardiometabolic risk factors (such as an increase in body mass index, hyperglycemia, hyperinsulinemia, dyslipidemia, hypertension, and metabolism-associated steatohepatitis)^19–21^ and actually precedes, in apparently still healthy individuals, the imminent advent of major cardiovascular events^20^ and the diagnosis of cancers including NSCLC.^22^ To establish causality in these patient-relevant statistical correlations, we performed preclinical experiments showing that antibody-mediated neutralization of DBI can reduce high fat diet-induced weight gain and diabetes,^23^ prevent or reverse steatohepatitis,^21,24^ combat myocardium infarction and heart failure,^20,24^ and improve immunosurveillance of various cancers including NSCLC.^22^ Thus, injections of neutralizing anti-DBI mAb reduce cancer progression as a monotherapy and enhance tumor growth control by anti-PD-1 mAb in murine models.^22^ Accordingly, anti-DBI mAb increases the infiltration of tumors by activated, non-exhausted cytotoxic T lymphocytes but reduces the infiltration of such tumors by regulatory T cells.^22,25^

As its name indicates, diazepam binding inhibitor (DBI) displaces the prototypic benzodiazepine diazepam from its cell surface receptor, which is a combination of specific subunits (alpha-1 and gamma-2) of the gamma-aminobutyric acid (GABA) A type receptor (GABAR_A_).^26^ A specific single amino acid substitution (F77I) in gamma-2 (gene/protein symbol: GABRG2) abolishes the binding of both DBI and diazepam to cells.^27–30^ Mice bearing this mutation in homozygosity (genotype: *Gabrg2*^*F77I/F77I*^) bear a similar phenotype as mice subjected to the conditional knockout of *Dbi* or receiving systemic injections of anti-DBI mAb.^24^ Importantly, administration of diazepam to mice fully reversed the beneficial effects of anti-DBI mAb on tumor progression in the context of chemoimmunotherapy,^31^ underscoring the functional relationship between the exogenous benzodiazepine and its endogenous equivalent (DBI or ‘endozepine’). In line with these preclinical results, we observed in a small French-Canadian cohort of NSCLC patients under immunotherapy targeting PD-1 or PD-L1 (*n* = 205) that benzodiazepine use correlated with reduced progression-free survival (PFS). Importantly, in this cohort, other psychotropic drugs than benzodiazepines did not affect PFS, suggesting a ‘specific’ effect of benzodiazepines.^31^ However, this cohort was too heterogeneous (e.g., because patients from two different countries received a range of antibodies targeting PD-1 or PD-L1) and too small (*n* = 205) to reach definitive conclusions.

For this reason, we decided to interrogate the French nation-wide ATHENA cohort, the largest real-world dataset on pembrolizumab treatment (*n* > 50,000),^8^ to identify possible effects of benzodiazepine medication on the clinical outcome of NSCLC patients treated with one single PD-1 antibody, pembrolizumab. In addition, we show that benzodiazepine use correlates with an unfavorable TOPOSCORE.

## Methods

### ATHENA study

#### Ethics and data protection

ATHENA is a retrospective cohort study using a comprehensive administrative database aimed firstly at exploring the optimal duration of pembrolizumab and secondly real-life prognosis factors in patients with advanced NSCLC.^8^ This study was conducted in accordance with the Declaration of Helsinki, as well as institutional and ethical rules concerning research regarding patient data. In accordance with French regulations applicable to the French National Health Insurance database (Système National des Données de Santé, SNDS), a strictly anonymous database,^32^ no informed consent was required. This study has been declared and approved prior to data extraction on the Health Data Hub online platform of the French data protection agency (Commission nationale de l’informatique et des libertés – CNIL, No. F20230713113749).

#### Study population

The database^33^ included (i) demographic data, (ii) hospital discharge reports, (iii) outpatient care, and (iv) long-term illness records. The cohort initially enrolled 50,083 patients with advanced stage metastatic non-small cell lung cancer treated with pembrolizumab. Among those 41,529 patients received pembrolizumab as first line (alone or under chemo-immunotherapy) between January 1, 2015, and December 31, 2022. Only patients who survived a minimum of 2 months after the initiation of pembrolizumab-based immunotherapy and without missing data were included in the analysis (*n* = 31,479) to study the possible effects of benzodiazepine exposure.

#### Concomitant benzodiazepine medication

Concomitant medications with benzodiazepines (indicated according to the World Health Organization Anatomical Therapeutic Chemical [ATC] classification as ATC N05BA) were identified from outpatient drug delivery data as a minimum of two prescriptions between day 90 before and day 30 after the first administration of pembrolizumab. This time interval was chosen because it corresponds to the one during which antibiotic use compromises the efficacy of immunotherapy in meta-analyses.^34,35^ ATC N05BA lists benzodiazepine derivatives including adinazolam, alprazolam, bentazepam, bromazepam, camazepam, chlordiazepoxide, clobazam, clotiazepam, cloxazolam, diazepam, fludiazepam, ethyl loflazepate, etizolam, halazepam, ketazolam, lorazepam, medazepam, mexazolam nordazepam, oxazepam, pinazepam, potassium clorazepate, prazepam, and tofisopam.

#### Statistical methods

We adjusted the population by means of a Cox model pondered by the Inverse Probability of Treatment Weighting (IPTW) score constructed with the following variables: sex, immunotherapy or chemoimmunotherapy, age, year of diagnosis, hospital type according to the volume of patient recruitment (high in upper decile, intermediate, or volume in the bottom 69%), full or day hospitalization, history of myocardial infarction, history of heart failure, history of stroke, history of liver disease, peripheral artery disease, chronic-obstructive pulmonary disease, history of renal failure, history of diabetes, use of antihypertensive drugs such as angiotensin converting enzyme and angiotensin receptor 2 antagonists, lipid-lowering agent, antiplatelet, anticoagulant, diuretic drugs, beta blockers, non-steroidal anti-inflammatory drugs, antipsychotics, antidepressants, thyroid hormone replacement, painkillers, opiate replacement therapy, exposure to antibiotics, proton pump inhibitors, corticosteroids, antiepileptic drugs, history of radiotherapy, as well as precariousness index of the municipality of residence (to correct for sociodemographic parameters). Confidence intervals were estimated with the bootstrap procedure. Time-to-event endpoints were estimated using the Kaplan–Meier method. All analysis was performed with R (v4.1.2).

### ONCOBIOTICS cohort

#### Ethics and study population

The Oncobiotics trial (NCTC04567446) is a multicenter study assessing the impact of the microbiome on outcomes in advanced NSCLC patients treated with anti-PD-(L)1 therapies, either alone or in combination with chemotherapy or tyrosine kinase inhibitors. Conducted across 14 centers in France and Canada, the study followed standard care until disease progression, intolerable side effects, or a maximum of 2 years of ICI treatment. Eligibility criteria and baseline data, including recent medications, are outlined in the trial protocol and recorded in electronic case reports.^14^ All participants provided written informed consent. GDPR and anonymization procedures were adhered to, in accordance with Oncobiome H2020 at ClinicObiome, Gustave Roussy. Data and sample collection complied with regulatory and ethical requirements, as well as ICH E6(R2) Good Clinical Practice (GCP) and the Declaration of Helsinki. Information on ancestry, race, ethnicity, and socioeconomic status was excluded due to France’s Data Protection Act No. 78–17.

#### Concomitant benzodiazepine and antibiotics medication

Concomitant medications with benzodiazepines (indicated according to the World Health Organization Anatomical Therapeutic Chemical [ATC] classification as ATC N05BA) and antibiotics (ATC J01) were identified from outpatient drug delivery data. We focused on patients with a minimum of two prescriptions within 60 days prior to the first administration of immune checkpoint inhibitors. This time interval was selected because it corresponds to the one during which antibiotic use has been shown to compromise the efficacy of immunotherapy in meta-analyses. Among 556 patients, stool samples were available from all participants before ICI initiation and, in some cases, after the use of antibiotics or benzodiazepines. These samples were used for shotgun metagenomics analysis to define microbiota composition (Table S1).

#### Microbiota analyses

Stool samples were processed for DNA extraction and sequencing using Ion Proton technology, following the MetaGenoPolis protocols. Prokaryotic taxonomic profiling at species level was performed using MetaPhlAn4 (vJan21_CHOCOPhlAn) (doi:https://doi.org/10.1038/s41587–023–01688-w). TOPOSCORE classification was based on publicly available algorithms (https://github.com/valerioiebba/TOPOSCORE), as previously published.^14^ Microbial alpha diversity (Shannon and Richness metrics) was computed with the vegan package in R environment. For beta diversity, statistical significance was determined using a PERMANOVA model, accounting for age, gender, body mass index (BMI), Eastern Cooperative Oncology Group (ECOG) performance status, antibiotics, and benzodiazepine (BZD) use. Discriminant species were identified using the Wilcoxon–Mann–Whitney test and by considering false discovery rate for multiple hypothesis testing correction.

#### Statistical methods

Progression free survival (PFS) was defined as time to progression or death and overall survival (OS) as the time to death, with patients censored at the last follow-up time if no event occurred. Survival analyses were performed using Kaplan–Meier curves, log-rank tests, and Cox regression, with significance defined as *p* < 0.05. Variables included in the multivariate model are reported in Supplemental Table S2. Least discriminant analysis effect size (LEfSe) analysis was used to determine discriminant species, which were identified using the Wilcoxon–Mann–Whitney test and by considering false discovery rate for multiple hypothesis testing correction.

## Results

### Pembrolizumab-treated lung cancer patients and benzodiazepine comedication

Our nation-wide database includes 50,083 patients with NSCLC that were treated with pembrolizumab. Among these patients, we only included those receiving pembrolizumab alone or together with chemotherapy in first line, who survived >2 months after treatment initiation and for which records on comedication were available to be able to measure the potential effect of benzodiazepine medication (*n* = 31,479). Among these 31,479 participants, 11,878 (37.7%) received at least two prescriptions of benzodiazepines within 90 days before and 30 days after treatment initiation. We focused on this interval because it has been pinpointed as particularly critical for the long-term impact of antibiotic medication on immunotherapy outcome.^34,35^ The characteristics of this population are listed in Table 1. Benzodiazepine users were more often women (40.9% vs 28.9% in non-users), treated with chemo-immunotherapy (69.6% vs 63.5% in non-users), exposed to antibiotics (47.9% vs 39.6% in non-users), to steroids (67.5% vs 54.1% in non-users), and to proton pump inhibitors (63.9% vs 47.7% in non-users).

**Table 1.** Patient’s characteristics.

	Receiving benzodiazepine (*N* = 11878)	Not receiving benzodiazepine (*N* = 19601)	Overall (*N* = 31479)
**Sex**			
Female	4858 (40.9%)	5668 (28.9%)	10526 (33.4%)
Male	7020 (59.1%)	13933 (71.1%)	20953 (66.6%)
**Age at diagnosis**			
Mean (SD)	63.1 (9.59)	65.6 (9.77)	64.7 (9.78)
**Type of hospital**			
High volume	4853 (40.9%)	8423 (43.0%)	13276 (42.2%)
Intermediate volume	5314 (44.7%)	8234 (42.0%)	13548 (43.0%)
Low volume	1711 (14.4%)	2944 (15.0%)	4655 (14.8%)
**Deprivation index**			
First quintile	1920 (16.2%)	3351 (17.1%)	5271 (16.7%)
Second quintile	2356 (19.8%)	4063 (20.7%)	6419 (20.4%)
Third quintile	2480 (20.9%)	4032 (20.6%)	6512 (20.7%)
Fourth quintile	2639 (22.2%)	4153 (21.2%)	6792 (21.6%)
Fifth quintile	2483 (20.9%)	4002 (20.4%)	6485 (20.6%)
**Strategy**			
Pembrolizumab alone	3605 (30.4%)	7164 (36.5%)	10769 (34.2%)
Pembrolizumab plus chemotherapy	8273 (69.6%)	12437 (63.5%)	20710 (65.8%)
**Type of first hospitalization**			
Day hospital	8685 (73.1%)	15233 (77.7%)	23918 (76.0%)
Inpatient hospitalization	3193 (26.9%)	4368 (22.3%)	7561 (24.0%)
**Exposition to antibiotics at baseline**			
No	6188 (52.1%)	11850 (60.4%)	18038 (57.3%)
Yes	5690 (47.9%)	7754 (39.6%)	13444 (42.7%)
**Exposition to steroid at baseline**			
No	3862 (32.5%)	8996 (45.9%)	12858 (40.8%)
Yes	8016 (67.5%)	10605 (54.1%)	18621 (59.2%)
**Exposition to PPI at baseline**			
No	4292 (36.1%)	10260 (52.3%)	14552 (46.2%)
Yes	7586 (63.9%)	9341 (47.7%)	16927 (53.8%)
**Diabetes at diagnosis**			
No	10593 (89.2%)	16878 (86.1%)	27471 (87.3%)
Yes	1285 (10.8%)	2723 (13.9%)	4008 (12.7%)
**Antipsychotic at diagnosis**			
No	11236 (94.6%)	19182 (97.9%)	30418 (96.6%)
Yes	642 (5.4%)	419 (2.1%)	1061 (3.4%)
**Antidepressant at diagnosis**			
No	8728 (73.5%)	17561 (89.6%)	26289 (83.5%)
Yes	3150 (26.5%)	2040 (10.4%)	5190 (16.5%)

### Impact of benzodiazepines on overall survival

**Figure 1.**
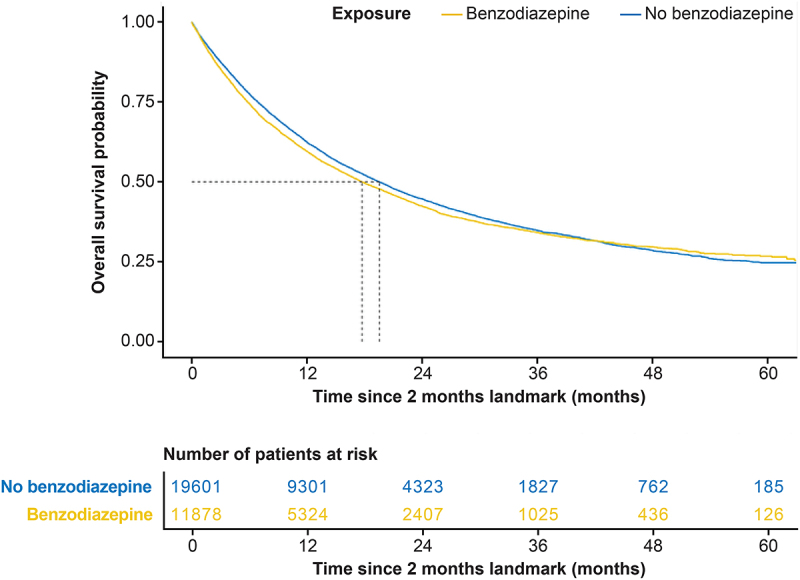
Impact of benzodiazepines on overall survival of lung cancer patient under pembrolizumab-based immunotherapy in the French national database including patients with non-small cell lung cancer that received immunotherapy. The graph compares the survival of patients after the 2 month-landmark that received benzodiazepines (orange line) or not (blue line). The number of surviving patients is indicated below the graph for selected time points.

As compared to control patients not receiving benzodiazepines (*n* = 19,601), benzodiazepine users (*n* = 11,878) exhibited diminished OS (Figure 1, hazard ratio [HR] = 1.08, 95% confidence interval [CI] 1.04 to 1.12). This statistically significant (*p* < 0.001) negative impact of benzodiazepine medication survived correction of the Cox model by means of an IPTW score pondering sociodemographic variables (age, sex, year of diagnosis, deprivation index), the history of arteriosclerotic, cardiometabolic, hepatic, renal or pulmonary disease, anticancer treatments including chemotherapy and radiotherapy, as well as other comedications with antibiotics, antihypertensive, antidiabetic, lipid-lowering, antiplatelet, anticoagulant, diuretic, beta receptor blocking, anti-inflammatory, analgesic, antipsychotic, or antidepressant drugs. This is particularly important, as previous studies have documented the negative effects of several of these variables on the OS of NSCLC cancer patients. Such observations apply to old age,^8,36^ male sex in the group of patients treated with chemoimmunotherapy,^8,36^ treatment in hospitals with a low volume of NSCLC cancer patients,^8^ residence in medically neglected areas,^8^ inpatient hospitalization during the first cycle of pembrolizumab treatment,^8^ past or present diabetes,^8^ treatment with antibiotics,^37^ diuretics,^8^ beta blockers,^8,38^ and painkillers including paracetamol.^8,39,40^

In conclusion, in this cohort, benzodiazepine use could be considered a negative prognostic factor for NSCLC patients treated with pembrolizumab, even after statistical correction for sociodemographic and health-related variables.

### Impact of benzodiazepines on the intestinal microbiota

**Figure 2.**
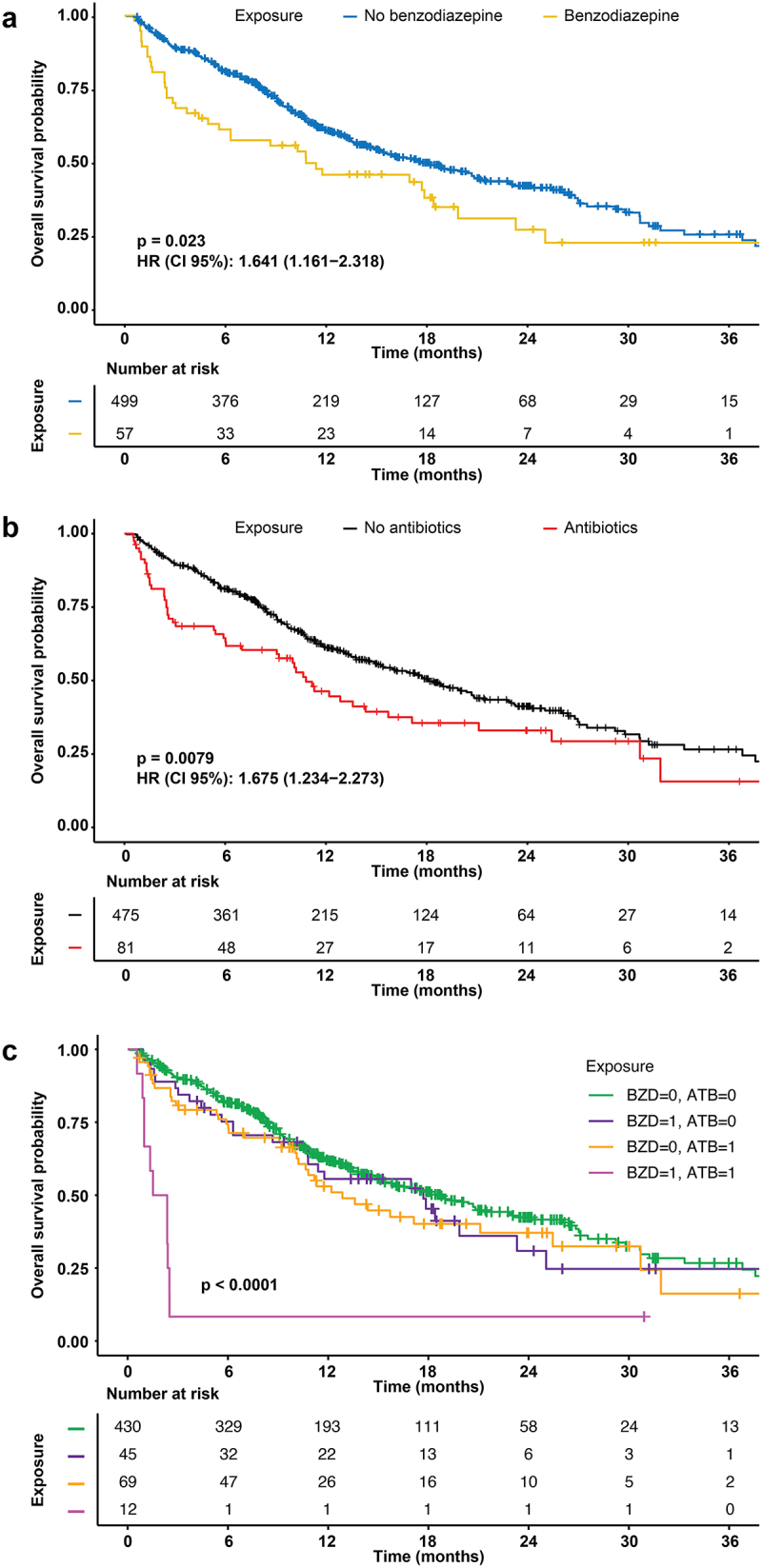
Impact of benzodiazepine and antibiotic use on overall survival of patients enrolled in the ONCOBIOTICS trial.

**Figure 3.**
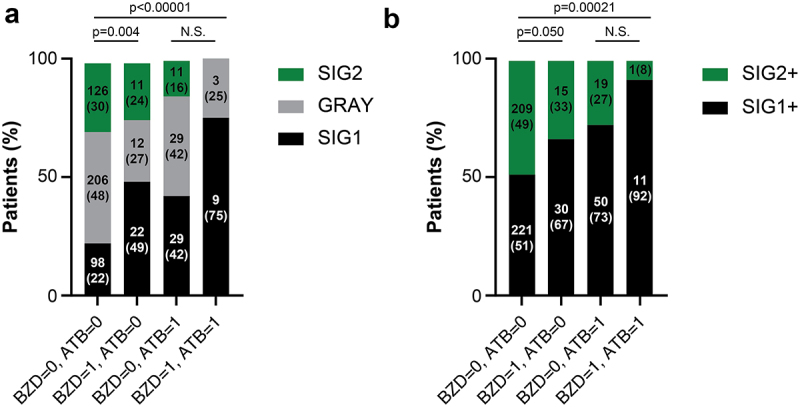
Impact of benzodiazepine and antibiotic use on the fecal microbiota in patients enrolled in the ONCOBIOTICS trial.

To investigate the impact of benzodiazepine use on the composition of the fecal microbiota, we analyzed data from our previous study (NCT03084471), which included microbial data from 556 patients with NSCLC. In this cohort, benzodiazepine use was associated with a significant reduction in OS (*p* = 0.023, Figure 2a), although effect size was smaller than that observed for the use of antibiotics (*p* = 0.008) in univariate analyses (Figure 2b). Indeed, in this relatively small cohort, benzodiazepine use was significantly associated with several confounding factors (Supplemental Table S1). Statistically, in this smaller cohort, the impact of benzodiazepines on OS did not remain significant in multivariate analyses (Supplemental Table S2). However, the combined use of benzodiazepines and antibiotics had a markedly stronger (and independent) negative effect on OS compared to either drug category alone (Figure 2c). The use of benzodiazepines had a significant effect on beta diversity after adjusting for age, gender, body mass index, and ECOG performance status (Supplemental Fig. S1A). When restricting the analysis to non-antibiotic-treated patients, a difference in fecal microbial composition was observed between patients consuming benzodiazepines compared to non-users, as determined by metagenomic shotgun sequencing and linear discriminant analysis effect size (LEfSe) analyses (Supplemental Fig. S1B). Indeed, in this comparative analysis, benzodiazepine use turned out to be associated with a significant increase in the abundance of four bacterial species belonging to the unfavorable species-interacting group (SIG) SIG1, but a decrease in species belonging to the favorable group SIG2. We recently demonstrated the prognostic value of classifying cancer patients based on their fecal microbiota into three groups: those with a predominantly unfavorable microbiota (SIG1), a mostly favorable microbiota (SIG2), or an intermediate (‘grey’) microbiota.^14^ Notably, among non-antibiotic users, benzodiazepine consumption was associated with a significant shift from SIG2 toward SIG1, a trend that was further exacerbated by antibiotic use (Figure 3a). Building on this observation, we calculated the TOPOSCORE, which combines information on the presence of bacterial species falling into the SIG1 and SIG2 categories with the abundancy of decisive *Akkermansia* species, to establish a dichotomic classification of cancer patients into SIG1+ and SIG2+ individuals. SIG1+ cancer patients under immunotherapy have a reduced OS compared to SIG2+ patients, as documented for NSCLC as well as other cancer types.^14^ Of note the SIG1/SIG2 prognostic ratio was increased in patients using both antibiotics and benzodiazepines (*p* = 0.00021) (Figure 3b).

(a) Impact of benzodiazepines (BZD) on overall survival (OS). (b) Impact of antibiotics (ATB) use on OS. (c) Combined use of antibiotics and benzodiazepines on OS. Use of benzodiazepines or antibiotics within the 60 days prior to treatment initiation is coded ‘1’, while the absence of prescription is coded ‘0’. *p*-values were calculated by means of the log-ranks (Cox–Mantel test). The number of surviving patients is indicated below the graph for selected time points.

(a) Classification of patients into three groups (SIG1, SIG2, Gray) according to their benzodiazepine and antibiotic use. (b) TOPOSCORE classification of patients into two groups (SIG1+, SIG2+). Use of benzodiazepines (BZD) or antibiotics (ATB) within the 60 days prior to treatment initiation is coded ‘1’, while the absence of prescription is coded ‘0’. Classifications were based on algorithms published in.^14^ Statistics were calculated by means of the chi-square test while replacing 0 values by 1. Absolute number of patients and relative percentages are displayed within bar plots without and within parentheses, respectively.

In conclusion, it appears that benzodiazepine use is associated with shifts in the fecal microbiota that are linked to poor immunotherapy outcome.

## Discussion

Our study suggests that benzodiazepines have a negative impact on NSCLC cancer immunotherapy outcome in a French nationwide cohort involving patients treated with pembrolizumab. Hence, this retrospective analysis supports the idea that benzodiazepines subvert immunosurveillance in NSCLC patients. Of note, as shown here, more than half of the patients of the ATHENA cohort were prescribed benzodiazepines during the critical time window (90 days before until 30 days after initiation of pembrolizumab immunotherapy), reflecting the highly prevalent use of this class of drugs in France.^41,42^ Current epidemiological estimates indicate that, in Western countries, 4–8% of the adult population regularly uses benzodiazepines,^43^ commensurate with the high level of addictiveness of this class of psychotropic drugs.^44,45^

Beyond the epidemiological association between benzodiazepine use and reduced efficacy of immunotherapy, the present study reveals a correlation between benzodiazepine consumption and stigmata of intestinal dysbiosis. The direction of causality between these two phenomena is still ambiguous since, on the one hand, oral benzodiazepine administration may affect the intestinal microbiota,^46,47^ and on the other hand, gut intestinal dysbiosis may affect immunity, general and mental health, hence increasing the need for benzodiazepine medication.^48^ Thus, future preclinical and clinical studies are needed to disentangle the potential mechanistic links between benzodiazepine use, dysbiosis, and poor immunotherapy outcome.

At this stage, we do not know whether the negative impact of benzodiazepines use would be independent of other established modulatory effects of the pembrolizumab response such as performance status, tumor burden, PD-L1 expression, tumor mutational burden, or gene expression signatures within the tumor^49^ (which all are not available in the SNDS database). In addition to cancer-related variables, other unmeasured confounders such as anxiety severity, insomnia, alcohol or tobacco consumption, food intake, physical activity, or the need for palliative care may influence the response to PD-1 therapy and hence were not corrected by our IPTW adjustment, If benzodiazepines had an immunosuppressive effect, one would expect that their prolonged use is associated with a surge of serious infectious diseases as well as cancer diagnosis. Indeed, there are preclinical and patient-centered epidemiological studies suggesting that this is the case.

The use of benzodiazepine has been linked to an increased risk of lung infections. Diazepam enhances the susceptibility of hamsters to respiratory infection with *Mycobacterium bovis*,^50^ as well as that of mice to infection with cowpox (CPXV), vaccinia virus (VACV),^51^ and *Streptococcus pneumoniae*.^52^ Accordingly, a meta-analysis of clinical studies found that benzodiazepine and benzodiazepine-related drugs increased risk of pneumonia among current users (with the most recent prescription within 30 days of the index data; OR = 1.4; 95% CI, 1.22–1.6), recent users (with the prescription within 31–90 days of the index date; OR = 1.38; 95% CI, 1.06–1.8) users, but not past users (prescription beyond 90 days; OR = 1.11; 95% CI, 0.96–1.27).^53^ This effect was particularly strong for the population younger than 65 years, with an increase in lung infections by 80%.^53^ Pneumonia has also been reported for hospitalized schizophrenic patients, in whom several benzodiazepines (such a diazepam, lorazepam, midazolam, and triazolam)^54^ and the benzodiazepine derivative clozapine^55,56^ were associated with increased risk.^57^

Of note, the effects of benzodiazepine are not limited to lung infection. Thus, diazepam sensitizes mice to intraperitoneal infection with *Salmonella typhimurium*.^58^ A retrospective population-based cohort study in South Korea revealed that long-term benzodiazepine use observed in 16 686 adult patients with sepsis was associated with increased 90-day mortality compared with propensity score-matched non-users (HR = 1.75, 95% CI 1.70–1.81).^59^ A recent study performed on the Swedish national registry focusing on the population under 65 years found that the use of benzodiazepines and related drugs (BZRD) was associated with an 83% relative increase in risk of serious viral or bacterial infections in any location of the body.^60^ This was found for 713,896 BZDR recipients compared to 713,896 age, sex, and residence-matched controls (HR = 1.83, 95% CI 1.79–1.89). The risk of serious infection was also found increased in a co-twin cohort involving 9197 BZRD-treated twins with their BZRD-untreated siblings (HR = 1.55, 95% CI 1.23–1.97) and when comparing 434,900 BZRD recipients to 428,074 patients taking selective serotonin reuptake inhibitors (HR = 1.33, 95% CI 1.30–1.35). The observed risks were similar across different types of BZDRs, with a dose–response association between cumulative BZRD dosage and risk of serious infections.^60^

Beyond their association with infectious diseases, prolonged benzodiazepine (ab)use has also been linked to an increase in the incidence of malignant disease. A pioneering study was conducted on a rural US population matching users of hypnotics with control subjects by sex, age ±5 years, smoking status, and start of period of observation. High-dose zolpidem ( > 800 mg/year, mean 3600, *N* = 1427) was found to be associated with higher cancer risk (HR = 1.28, 95% CI 1.03–1.59), and this was also observed for two dose levels of temazepam, an intermediate dose (240–1640 mg/year, mean 683, *N* = 613; HR = 1.44, 95% CI 1.05–1.98) and a high dose ( > 1640 mg/year, mean 7777, *N* = 665; HR = 1.99; 95% CI 1.57–2.52).^61^ A population-based retrospective analysis matching 59,647 benzodiazepine users with controls by age and sex revealed an increase in cancer risk in Taiwan. This study found an increase in overall cancer risk (HR = 1.19, 99.6% CI 1.08–1.32) and in particular liver cancer (HR = 1.45; 99.6% CI 1.10–1.90), prostate cancer (HR = 1.72, 99.6% CI 1.10–2.70), and bladder and kidney cancer (HR = 1.76, 99.6% CI 1.16–2.67).^62^ In the Danish nationwide registers, 152 510 cases with a first-time cancer who were matched (1:8) by age and gender to 1,220,317 cancer-free controls. In this study, long-term use of BZRD was defined by a cumulative amount of ≥500 defined daily doses of BZRD within a period of 1–5 years prior to the index date. The adjusted odds ratio (OR) of long-term BZRD for all cancers was 1.09 (95% CI 1.04–1.14). ORs were particularly high in descending order for liver (OR = 1.81, 95% CI 1.18–2.80), esophagus (OR = 1.43, 95% CI 1.01–2.02), stomach (OR = 1.40, 95% CI 1.05–1.88), kidney (OR = 1.39, 95% CI 1.01–1.91), lung (OR = 1.38, 95% CI 1.23–1.54) and pancreas (OR = 1.35, 95% CI 1.02–1.79).^63^ A later study on the same cohort included 94 923 patients with cancer and 759 334 age- and sex-matched (1:8) population controls, then implemented propensity score (PS) calibration to eliminate confounding factors and concluded similarly that benzodiazepine use was associated with an increased cancer risk (OR = 1.09, 95% CI 1.00–1.19).^64^

A meta-analysis of observational studies including a total of 18 case–control studies and 4 cohort studies (213,823 patients with cancer and 1,683,780 controls) found that benzodiazepine use was significantly associated with a dose-dependent increased risk of all cancers (pooled OR or relative risk [RR] 1.19, 95% CI 1.16–1.21). This effect was particularly strong – in descending order – for brain cancer (OR/RR = 2.08, 95% CI 1.77–2.44), esophagus cancer (OR/RR = 1.55, 95% CI 1.30–1.85), pancreatic cancer (OR/RR = 1.39, 95% CI 1.17–1.64), renal cancer (OR/RR = 1.30, 95% CI 1.14–1.49), prostate cancer (OR/RR = 1.26, 95% CI 1.16–1.37), liver cancer (OR/RR = 1.22, 95% CI 1.13–1.31), and lung cancer (OR/RR = 1.20, 95% CI 1.12–1.28).^65^ Another meta-analysis came to a similar conclusion (RR = 1.25, 95% CI 1.15–1.36), again revealing the highest risk for brain cancer (RR = 2.06, 95% CI 1.76–2.43),^66^ perhaps reflecting the fact that benzodiazepines have been designed to cross the blood–brain barrier and to accumulate in the central nervous system to exert their anxiolytic and sedative effects.^67^

Altogether, multiple retrospective studies suggest immunosuppressive effects of benzodiazepines that increase the incidence of a wide array of infectious and malignant diseases. The present study extends this conclusion to cancer immunotherapy that, in the case of NSCLC and its treatment by the anti-PD-1 antibody pembrolizumab, apparently is compromised by benzodiazepines and associated with intestinal dysbiosis. It will be important to broaden this kind of exploration to other cancer types and other modalities of immunotherapy including PD-L1 or CTLA-4 blockade, CAR-T cells, oncolytic viruses, or therapeutic vaccination.

### Limitations of the study

The present study reports a statistical association between death from any cause and the use of benzodiazepine over the period studied in very large cohort ( >50 000 patients). Hence, the study does not explore disease-specific survival (rather than overall survival) and only evaluates benzodiazepine use within a limited time window. Although it appears plausible that, given their high level of addictiveness, benzodiazepine use is continued in most of the individuals, no information on possible cumulative effects has been retrieved. Moreover, patients with more advanced NSCLC or more severe comorbidities could be particularly exposed to benzodiazepines. The ATHENA cohorts do not report major factor that influence immunotherapy benefit such as performance status, PD-L1 expression, tumor burden, presence of liver metastases, or tumor mutational burden. The need for an anxiolytic treatment could be related to a more symptomatic disease, reflecting a higher tumor burden and a worse outcome. The mechanisms through which benzodiazepines cause immunosuppression, which may include intestinal dysbiosis, require further experimental and clinical exploration.

Irrespective of these limitations, the available data from this and other cohort studies indicate that benzodiazepine (ab)use by NSCLC patients under PD-1/PD-L1 blockade is associated with reduced PFS, OS, and even immune-related adverse events, in line with general immunosuppressive effects of this class of psychotropic drugs.

## Supplementary Material

Table S1.docx

Table S2.docx

## Data Availability

Individual patient data cannot be shared due to protection of privacy, but access to the ATHENA cohort can be requested to the National Health Data System on the French Data Hub platform. Metagenomics data from the ONCOBIOTICS will be made available upon reasonable request.
