# Supplementary material for: Benzodiazepines interfere with the efficacy of pembrolizumab-based cancer immunotherapy. Results of a nationwide cohort study including over 50,000 participants with advanced lung cancer
Source: Oncoimmunology. 2025 Jul 4;14(1):2528955. doi: 10.1080/2162402X.2025.2528955 (PMC12233713; doi:10.1080/2162402X.2025.2528955)
Supplement: Table S1.docx [file KONI_A_2528955_SM0936.docx]

|  |  | **All patients**  **(n=556)** | **BDZ**  **(n=57)** | **noBDZ**  **(n=499)** | **p-value**  **(Chi square)** |
| --- | --- | --- | --- | --- | --- |
|  |  |  |  |  |  |
| **Sex – No (%)** | **Male** | 202 (36) | 25 (44) | 177 (36) | *0.25* |
|  | **Female** | 354 (64) | 32 (56) | 322 (64) |  |
| **Age (year)** | **Median (range)** | 64 (24-92) | 64 (32-85) | 66 (24-92) | ***0.02**** |
| **BMI, (kg/m^2^) – No (%)** | **<18** | 34 (6) | 8 (15) | 26 (5) | ***0.03*** |
|  | **[18-25]** | 301 (56) | 31 (56) | 270 (56) |  |
|  | **≥25** | 207 (38) | 16 (29) | 191 (39) |  |
|  | **Unknown** | 14 | 2 | 12 |  |
| **ECOG performance status - No (%)** | **0-1** | 449 (86) | 43 (78) | 406 (87) | *0.10* |
|  | **≥2** | 73 (14) | 12 (22) | 61 (13) |  |
|  | **Unknown** | 34 | 2 | 32 |  |
| **Treatment(s) line(s) - No (%)** | **1** | 205 (37) | 23 (40) | 182 (37) | *0.57* |
|  | **≥2** | 351 (63) | 34 (60) | 317 (63) |  |
| **Treatment type - No (%)** | **IO** | 485 (87) | 5 (9) | 433 (87) | ***<0.01*** |
|  | **IO/CT** | 67 (12) | 52 (91) | 62 (12) |  |
|  | **IO/Other agent** | 4 (1) | 0 (0) | 4 (1) |  |
| **Antibiotics -60/0 days - No (%)** | **Yes** | 81 (15) | 12 (21) | 69 (14) | *0.16* |
|  | **No** | 475 (85) | 45 (79) | 430 (86) |  |
| BDZ: benzodiazepine; BMI : Body Mass Index ; ATB : Antibiotics ; IO: Immunotherapy ; CT : Chemotherapy ;  *Wilcoxon rank sum test | | | | | |
