# Supplementary material for: Benzodiazepines interfere with the efficacy of pembrolizumab-based cancer immunotherapy. Results of a nationwide cohort study including over 50,000 participants with advanced lung cancer
Source: Oncoimmunology. 2025 Jul 4;14(1):2528955. doi: 10.1080/2162402X.2025.2528955 (PMC12233713; doi:10.1080/2162402X.2025.2528955)
Supplement: Table S2.docx [file KONI_A_2528955_SM0935.docx]

| **Table** | | | | | | | | |
| --- | --- | --- | --- | --- | --- | --- | --- | --- |
| **Variables** | **Univariate analysis** | | | | **Multivariate analysis¹** | | | |
|  | **Groups** | **Hazard ratio** | **Confidence interval 95%** | **p-value** | **Groups** | **Hazard ratio** | **Confidence interval 95%** | **p-value** |
| **Benzodiazepine** | No (n=499) | Reference | | | No (n=499) | Reference | | |
|  | Yes (n=57) | 1.490 | 1.054-2.105 | **0.024** | Yes (n=57) | 1.365 | 0.926-2.01 | 0.116 |
| **BMI** | <18 (n=34) | Reference | |  | <18 (n=34) | Reference | | **0.002** |
|  | [18-25] (n=301) | 0.780 | 0.500-1.220 | 0.277 | [18-25] (n=301) | 0.939 | 0.583-1.506 | 0.793 |
|  | ≥25 (n=207) | 0.520 | 0.326-0.830 | **0.006** | ≥25 (n=207) | 0.609 | 0.370-1.002 | **0.051** |
| **Antibiotics (-60/0 days)** | No (n=475) | Reference | | | No (n=475) | Reference | | |
|  | Yes (n=81) | 1.509 | 1.111-2.049 | **0.008** | Yes (n=81) | 1.656 | 1.19-2.27 | **0.002** |
| **ECOG-PS** | 0-1 (n=449) | Reference | | | 0-1 (n=449) | Reference | | |
|  | 2-4 (n=73) | 1.733 | 1.271-2.362 | **<0.001** | 2-4 (n=73) | 1 286 | 0.857-1.928 | 0.224 |
| **Treatment(s) line(s)** | 1 (n=205) | Reference | | | 1 (n=205) | Reference | | |
|  | ≥2 (n=351) | 1.678 | 1.276-2.206 | **<0.001** | ≥2 (n=351) | 1.801 | 1.315-2.468 | **<0.001** |
| **Age, per year (median range)** | n=556 | 1.00 | 0.986-1.01 | 0.811 | n=556 | 1.00 | 0.99-1.01 | 0.933 |
| **Treatment type** | IO (n=485) | Reference | |  | IO (n=485) | Reference | | |
|  | IO/CT (n=67) | 0.681 | 0.431-1.08 | 0.100 | IO/CT (n=67) | 0.980 | 0.588-1.637 | 0.940 |
|  | IO/Other agent (n=4) | 1.049 | 0.260-4.219 | 0.947 | IO/Other agent (n=4) | 1.548 | 0.380-6.291 | 0.541 |
| **Gender** | Male (n=354) | Reference | | | Male (n=354) | Reference | | |
|  | Female (n=202) | 0.838 | 0.655-1.07 | 0.158 | Female (n=202) | 0.89 | 0.68-1.15 | 0.377 |
| ¹Total patients included=556; missing data: 48; total events: 508 | | | | | | | | |
